# Highly Stable Sn─Pb Perovskite Solar Cells Enabled by Phenol‐Functionalized Hole Transporting Material

**DOI:** 10.1002/anie.202424515

**Published:** 2025-04-02

**Authors:** Jianchang Wu, Manman Hu, Qingqing Dai, Ecem Aydan Alkan, Anastasia Barabash, Jiyun Zhang, Chao Liu, Jens A. Hauch, Gao‐Feng Han, Qing Jiang, Tonghui Wang, Sang Il Seok, Christoph J. Brabec

**Affiliations:** ^1^ Forschungszentrum Jülich GmbH Helmholtz‐Institute Erlangen−Nürnberg (HI‐ERN) 91058 Erlangen Germany; ^2^ Faculty of Engineering Department of Material Science Materials for Electronics and Energy Technology (i‐MEET) Friedrich‐Alexander‐Universität Erlangen−Nürnberg (FAU) 91058 Erlangen Germany; ^3^ Department of Energy Engineering School of Energy and Chemical Engineering Ulsan National Institute of Science and Technology (UNIST) Ulsan 44919 South Korea; ^4^ Key Laboratory of Bionic Engineering Ministry of Education Jilin University Changchun 130022 China; ^5^ Key Laboratory of Automobile Materials (Jilin University) Ministry of Education and School of Materials Science and Engineering Jilin University Changchun 130022 China; ^6^ Zernike Institute for Advanced Materials University of Groningen Groningen 9747 AG the Netherlands

**Keywords:** Alcohol soluble, Hole transporting material, Phenol‐functionalized, Sn─Pb perovskite solar cells, Tandem solar cells

## Abstract

Sn─Pb perovskites, a most promising low bandgap semiconductor for multi‐junction solar cells, are often limited by instability due to the susceptibility of Sn^2+^ to oxidation. Inspired by the antioxidative properties of polyphenolic compounds, we introduce the reductive phenol group and strong electronegative fluorine into an organic conjugated structure and design a multi‐functional polymer with fluorine and phenol units (PF─OH). The design of PF─OH allows the effective rise in the energy barrier of Sn^2+^ oxidation, leading to a significant enhancement in the stability of Sn─Pb perovskite devices from 200 to 8000 h—an improvement of around 100 times. Additionally, the strong binding energy between Sn^2+^ and the phenol in PF─OH critically influences Sn─Pb perovskite's crystallization and grain growth, resulting in perovskite films with fewer pinholes at the buried interface and extended carrier lifetimes. This enhancement not only boosts the power conversion efficiency (PCE) to 23.61%, but also significantly improves the operational stability of the devices. Ultimately, this design strategy has been proven universal through the phenolization of a series of molecules, marking a milestone in enhancing the stability of Sn─Pb perovskites.

## Graphical abstract

Stability is the key challenge for Sn─Pb perovskites. This work tackles the critical stability issue by introducing phenol hydroxylation in the hole transporting material (HTM). The phenol group enhances oxidation resistance and regulates crystallization kinetics, while fluorine improves energy alignment. This strategy boosts device stability by 40 times and achieves a 23.61% power conversion efficiency (PCE), offering a universal approach for advancing Sn─based perovskites.

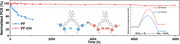

## Introduction

Halide perovskites are emerging as standout materials for next‐generation solar cells due to their excellent photovoltaic properties and cost‐efficient production.^[^ ^1^, ^2^, ^3^, ^4^, ^5^, ^6^, ^7^ ^]^ Notably, pure lead‐based perovskite solar cells (PSCs) have achieved a record‐breaking power conversion efficiency (PCE) of over 26%, rivaling traditional silicon solar cells.^[^ ^6^, ^8^ ^]^ However, despite their impressive performance, lead‐based perovskites encounter substantial challenges, including environmental concerns due to the toxicity of lead^[^ ^9^, ^10^, ^11^, ^12^ ^]^ and a slightly too large bandgap of 1.55 eV^[^ ^9^ ^]^ for all perovskite multijunction solar cells. These limitations have spurred interest in developing alternative perovskite materials with fewer drawbacks. Among these, Sn─Pb perovskites show promise with their adjustable, narrower bandgap and reduced environmental impact.^[^ ^13^, ^14^ ^]^

However, the integration of Sn^2+^ in perovskite structures incurs new challenges. The easy oxidation of Sn^2+^ and rapid crystallization lead to inferior efficiency and stability compared to Pb‐based counterparts.^[^ ^1^, ^9^, ^10^, ^15^ ^]^ Oxidation of Sn^2+^ occurs at every stage, including in precursor solutions, during film formation, and within operational devices.^[^ ^16^, ^17^ ^]^ Although incorporating Sn or Pb metal powders can effectively reduce Sn^4+^ in precursor solutions, they cannot be incorporated into films, thus failing to protect against later‐stage oxidation.^[^ ^18^, ^19^, ^20^, ^21^ ^]^

Organic antioxidants, widely applied in biological and semiconductor fields, might offer a solution. For example, vitamin C, a well‐known natural antioxidant, plays a crucial role in efficiently scavenging reactive oxygen species in living systems^[^ ^22^ ^]^ and organic semiconductors.^[^ ^2^ ^]^ Similarly, phenolic^[^ ^23^ ^]^ and hydrazine‐based^[^ ^24^, ^25^ ^]^ materials have been used in Sn‐based perovskites to inhibit Sn^2+^ oxidation. However, these antioxidants or passivators may induce the formation of 2D perovskites, potentially affecting device performance and stability.

In this study, we address these issues by introducing the phenol group into hole transporting materials (HTMs). We design a multi‐functional HTM polymer with fluorine and phenol units (PF─OH) specifically for Sn‐based PSCs. The carbazole and triphenylamine moieties in PF─OH act as strong electron‐donating groups, ensuring the molecule's *p*‐type characteristics and charge transport properties.^[^ ^26^, ^27^ ^]^ Additionally, the introduction of fluorine atoms enhances interactions with perovskite materials.^[^ ^28^ ^]^ Unlike traditional HTMs, the phenol units in our design intrinsically protect Sn^2+^ against oxidation most effectively throughout the perovskite formation process—before, during, and after film formation—and thus significantly lower the density of nonradiative recombination centers. Moreover, the strong binding between the phenol groups and SnI_2_ moderates the rapid crystallization of Sn‐based perovskites. Leveraging these systematic effects, high‐performance Sn─Pb PSCs utilizing PF─OH have achieved an impressive PCE of 23.61% and exhibit notably improved shelf storage stability, retaining 95% of their initial efficiency after 8000 h in a nitrogen atmosphere without encapsulation.

## Results and Discussion

### Molecular Design and Density Functional Theory (DFT) Analysis

**Figure 1:**
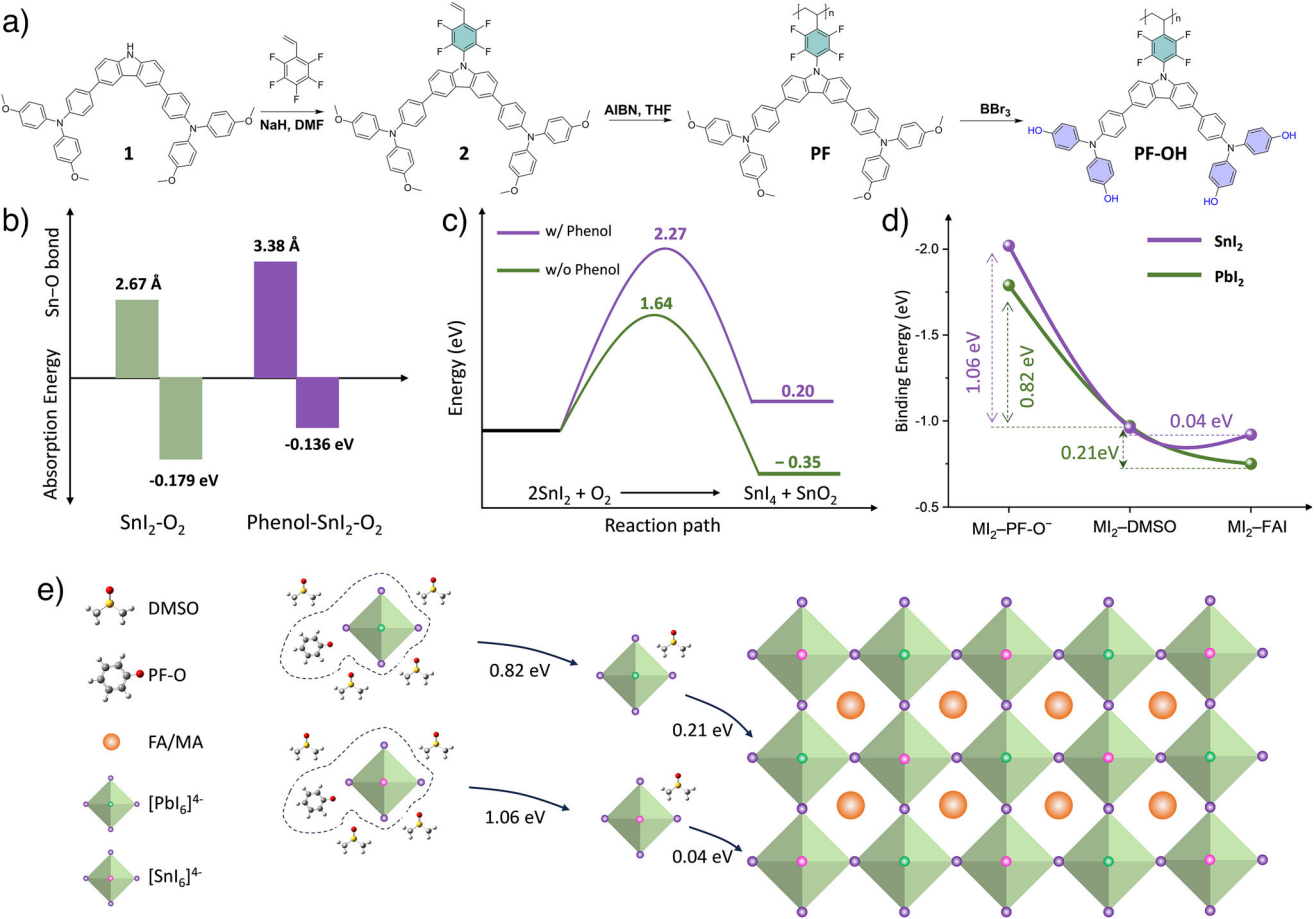
Molecular design to achieve equilibrium crystallization and suppress Sn^2+^ oxidation. a) Synthetic route of PF─OH. b) Schematic of the reduction in the binding energy of metal iodides (MI_2_)‐PF─O^−^, MI_2_‐DMSO, and MI_2_‐FAI using DMSO as solvent. PF─O^−^ is the product formed after the proton transfer of PF─OH. c) The adsorption energy of O_2_ on SnI_2_ and the Sn─O bond length with and without phenol. d) SnI_2_ oxidation process and reaction energy barrier. e) Schematic of PF─O^−^‐induced equilibrium crystallization in mixed Sn─Pb perovskites.

Inspired by our goal of stabilizing Sn─Pb PSCs, we aimed to synthesize an HTM polymer, poly‐4,4′,4″,4′′′‐(((9‐(2,3,5,6‐tetrafluoro‐4‐vinylphenyl)‐9*H*‐carbazole‐3,6‐diyl)bis(4,1‐phenylene))bis(azanetriyl))tetraphenol (PF─OH) with the following characteristics: first, to stabilize Sn^2+^, the normally used methoxy substitution (OMe) is replaced with a phenol group (PhOH) by a demethylation reaction. Second, to create semiconducting *p*‐type characteristics and suitable hole transport properties, carbazole and triphenylamine building blocks are selected as the main conjugated chain. Third, to finely tune the molecule's electronic structure and enhance the interaction with perovskite semiconductors, a fluorination strategy is adopted in this work. PF─OH was synthesized as outlined in Figure 1a. The conjugated backbone, compound **1**, was obtained by coupling dibromocarbazole with triphenylamine borate via a Suzuki coupling reaction.^[^ ^29^ ^]^ Subsequently, the fluorinated polymerizable monomer, compound **2**, was synthesized by nucleophilic substitution reaction of compound **1** with pentafluorostyrene, catalyzed by a strong base. The polymer, poly‐4,4′‐(9‐(2,3,5,6‐tetrafluoro‐4‐vinylphenyl)‐9*H*‐carbazole‐3,6‐diyl)bis(*N*,*N*‐bis(4‐methoxyphenyl)aniline) (PF), was then polymerized under the catalysis of azobisisobutyronitrile (AIBN). BBr_3_ was added to the polymer solution to convert methoxy groups to hydroxy groups to carry out a demethylation reaction, yielding the target product PF─OH.

To validate our molecular design, we conducted a DFT analysis to investigate the mechanisms that suppress Sn^2+^ oxidation and facilitate equilibrium crystallization. The initial oxidation stage involves the adsorption of oxygen molecules on Sn^2+^, which subsequently accepts electrons to form Sn^4+^. Our computational simulations provide insight into how phenol incorporation affects those two processes. The introduction of phenol increases the Sn ─O bond length in the absorbed state from 2.67 to 3.38 Å (Figure 1b). This makes the adsorption of oxygen on phenol‐bearing SnI_2_ less favorable and destabilizes the SnI_2_‐O_2_‐phenol complex. Further analysis revealed that the presence of phenol shifts the reaction (Sn^2+^ − 2e → Sn^4+^) from thermodynamically spontaneous (reaction enthalpy, Δ*H* = −0.35 eV) to non‐spontaneous (Δ*H* = 0.20 eV) (Figure 1c). Calculations of the transition state show that the activation energy increases from 1.64 to 2.27 eV in the presence of phenol, indicating that higher energy is required to activate the reaction. These data suggest that phenol groups inhibit oxygen adsorption on the surface of SnI_2_ and raise both the reaction's activation energy and Gibbs free energy, thereby effectively curbing the oxidation process.

The binding energies of various phases were calculated to analyze the crystallization kinetics of Sn─Pb perovskite. In Pb‐based perovskites, dimethyl sulfoxide (DMSO) is commonly employed to regulate the crystal growth rate due to its strong binding with PbI_2_. Perovskite formation does not proceed directly from PbI_2_ reacting with formamidinium iodide (FAI) but through an intermediate, PbI_2_·DMSO, reacts with FAI, which moderates crystal growth.^[^ ^30^, ^31^, ^32^ ^]^ However, for Sn‐based perovskites, the effectiveness of DMSO is minimal.^[^ ^17^ ^]^ Our findings reveal that the binding energy differences between PbI_2_‐DMSO and PbI_2_‐FAI are substantial at 0.21 eV, but they narrow down to just 0.04 eV when PbI_2_ is replaced with SnI_2_ (Figure 1d). This indicates that, thermodynamically, SnI_2_‐DMSO has no obvious advantage over SnI_2_‐FAI and explains the ineffectiveness of DMSO in regulating crystallization rates in tin‐based perovskites. When PF─O^−^ is involved, its interaction with metal iodides is markedly stronger than that of DMSO. Remarkably, it forms the highest binding energy with SnI_2_, suggesting that the intermediate complexes formed with SnI_2_ are more stable. This delays the precipitation and nucleation of a perovskite phase, thereby decelerating the crystallization process in Sn─Pb mixed perovskites more effectively (Figure 1e).

### Material Properties

After demethylation, a significant increase in molecular polarity is observed, rising from 1.83 to 2.85D (Figure S4). This change results in poor solubility in low‐polarity solvents like chloroform and chlorobenzene while significantly improving solubility in alcoholic solvents, where it exceeds 10 mg mL^−1^. Demethylation has minimal impact on the molecular conjugated structure. PF─OH exhibits an absorption peak at 360 nm, similar to PF, which aligns with the absorption characteristics of structurally similar compounds.^[^ ^29^, ^33^ ^]^ Distinct from PF, however, PF─OH displays a broad and weak absorption peak in the 450 to 600 nm range (Figure S5). This is attributed to the active protons on the phenol groups, which likely undergo proton transfer to adjacent nitrogen atoms, resulting in self‐doping.^[^ ^34^, ^35^, ^36^ ^]^ This phenomenon is evident in the color variation between the two compounds: PF appears pale yellow, while PF─OH is cyan‐green. Doping typically enhances the charge density within the material, significantly improving mobility.^[^ ^35^, ^37^, ^38^ ^]^ Space‐charge limited current (SCLC) measurement demonstrates that the mobility of the polymer increases from 5.75 × 10^−5^ to 2.5 × 10^−4^ cm^2^ V^−1^ s^−1^ following demethylation (Table 1 and Figures S5 and S6). Additionally, the glass transition temperature (*T*_g_) of the material increases from 121° to 148 °C after demethylation, owing to the enhanced intermolecular hydrogen bonds, improving the thermal stability of the device. Cyclic voltammetry (CV) was performed to investigate the redox behaviors of the polymers and calculate the highest occupied molecular orbital (HOMO) levels. Compared to PF, PF─OH has a lower oxidation potential, around 0.14 V (0.22 V for PF), indicating a higher HOMO level for PF─OH, which is inconsistent with the DFT results. The calculated HOMO level of PF─OH is −5.24 eV, which meets the requirements for perovskite applications. In addition, PF─OH displays a smaller contact angle (73.7°) than PF (93.5°) and PEDOT:PSS (poly(3,4‐ethylenedioxythiophene) polystyrene sulfonate, 87.6°), suggesting a better wettability of the perovskite precursor on PF─OH, facilitating a uniform coverage of the perovskite film (Figure S8). Moreover, PF─OH can minimize the parasitic absorption loss of PEDOT:PSS, leading to enhanced transmittance in the range of 600–1200 nm for the FTO/PF─OH sample (Figure S9).

**Table 1:** Optical, electrochemical, and charge transport properties of PF and PF─OH

HTMS	*λ*_max,sol_ (nm)	*E*_g_ ^a)^ (ev)	*E* ^ox^_onset_ (v)	*E*_HOMO_ ^b)^ (ev)	*µ*_h_ ^c)^ (cm^2^ v^−1^ s^−1^)	Contact angle^d)^	*T_g_* (°C)
PF	406	3.05	0.22	−5.32	5.75 × 10^−5^	93.5	121
PF─OH	406, 551	3.05	0.14	−5.24	2.51 × 10^−4^	73.7	148

^a)^ Estimated from the absorption onset wavelength, *E*_g_ (eV) = 1240/*λ*_onset_ (nm);

^b)^ Calculated from *E*_HOMO_ = −(*E* ^ox^_onset_ + 5.10) (eV);

^c)^ Hole mobilities measured by SCLC method with structure: FTO/PEDOT:PSS/HTMs/MoO_3_/Ag;

^d)^ The contact angle of perovskite precursor on HTMs.

**Figure 2:**
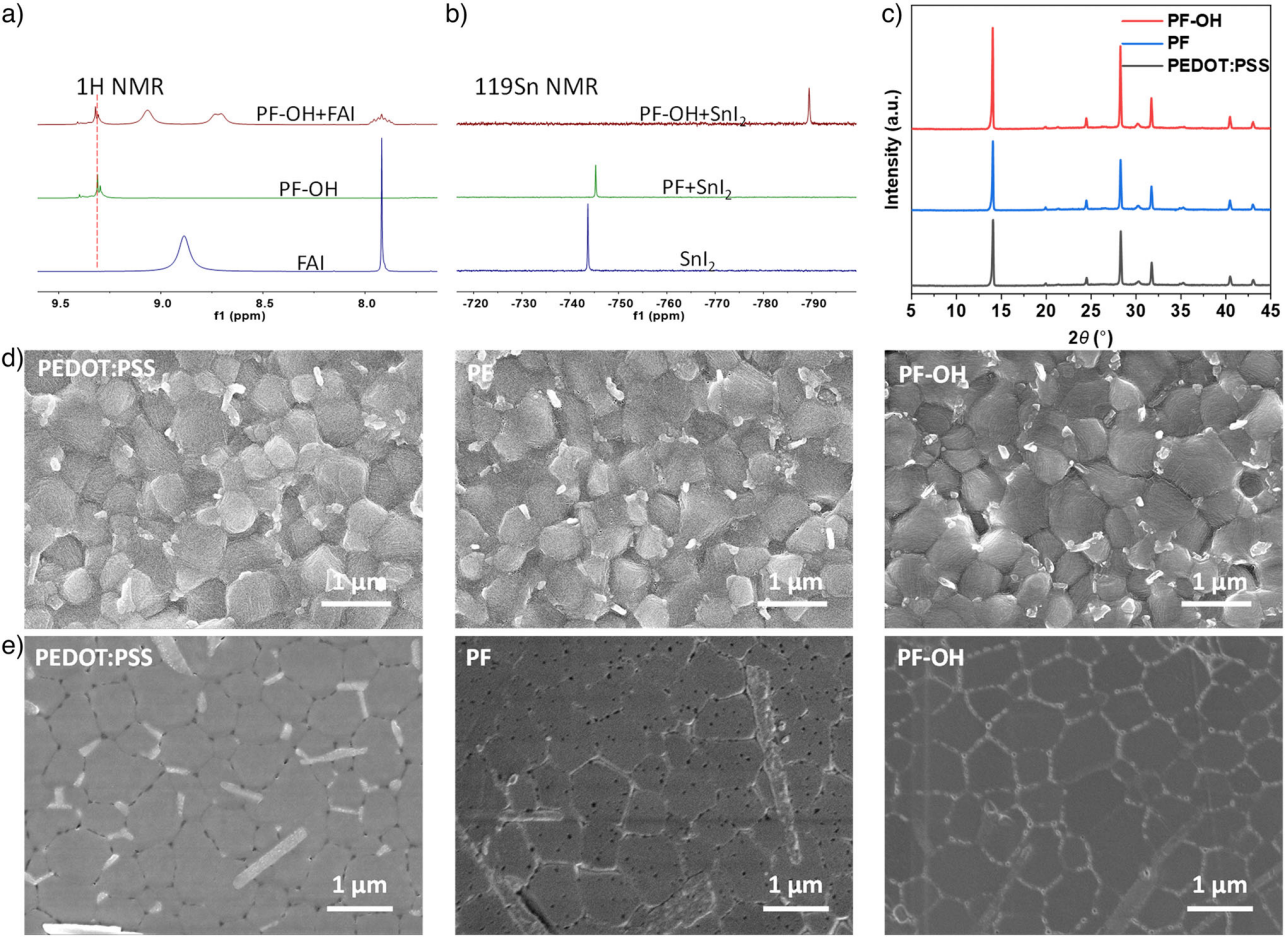
Influence of HTMs on perovskite film and buried interface. a) ^1^H NMR of FAI, PF─OH, and PF─OH + FAI mixture in DMSO‐*d6*. b) ^119^Sn NMR of SnI_2_, PF + SnI_2_ mixture, and PF─OH + SnI_2_ mixture in DMSO‐*d6*. c) XRD patterns of perovskite films grown on different HTMs. d) The top surface of perovskite films grown on different HTMs. e) The buried interface of perovskite films grown on different HTMs.

To explore the effects of phenolic hydroxyl groups on perovskite following demethylation, liquid‐state hydrogen proton and tin nuclear magnetic resonance (^1^H, ^119^Sn NMR) were conducted. We found that PF─OH has intermolecular interactions with perovskite precursor constituents, including SnI_2_ and formamidinium iodide (FAI) (Figure 2a). It can be observed that the ═NH_2_ proton peaks (*δ* 8.89 ppm) of FAI split into two peaks, while the CH proton peaks (*δ* 7.92 ppm) shift from a singlet to a septet, likely due to hydrogen bond formation between PF─OH and FAI. This hydrogen bond suppresses the rapid proton exchange of ═NH₂, creating distinct chemical environments. Additionally, the peak (*δ* 9.32 ppm) corresponding to the ─OH group in PF─OH also shifts slightly downfield, further supporting the presence of hydrogen bond interactions. After adding PF─OH, significant shifts in 119Sn NMR towards high field suggest that the electrons transfer from PF─OH to Sn^2+^ (Figure 2b). In contrast, the addition of PF has a minimal effect on Sn NMR. This suggests that the strong interaction between PF─OH and the perovskite is primarily due to the presence of phenol hydroxyl groups. Additionally, due to its increased polarity, PF─OH exhibits suitable solubility in polar solvents such as *N,N*‐dimethylformamide (DMF) and DMSO, allowing a small amount of PF─OH to dissolve in the perovskite precursor during the spin‐coating process. This phenomenon was confirmed by washing a PF─OH film with a DMF: DMSO mixture and observing changes in absorption (Figure S10). To further verify the presence of trace amounts of PF─OH within the film, we prepared a perovskite on a thick PF─OH film. The top layer of the perovskite film was gently scraped off. To avoid removing the underlying PF─OH, we ensured that a visible layer of perovskite film remained on the substrate after scraping. Following the removal of the film and then testing with ^1^H NMR, the presence of trace amounts of PF─OH was detected (Figure S11). This evidences that PF─OH functions not only at the buried interface but also extends its effects throughout the interior of the perovskite film. We then calculated the binding energies of the ─OMe and ─OH groups with the undercoordinated Sn^2+^ or Pb^2+^ in perovskites (Figure S12), considering that those groups exhibit a more negative electrostatic surface potential in the molecules. It is found that PF─OH has a stronger coordination ability with SnI_2_ (−0.94 eV) and PbI_2_ (−0.98 eV) than PF (−0.88 eV for SnI_2_, −0.93 eV for PbI_2_). Considering the self‐doping and the proton transfer from the phenolic hydroxyl groups on PF─OH to the alkaline groups of FAI and MAI, part of the molecules exists as oxyanions, specifically PF─O^−^. We further calculated the interaction of PF─O^−^ with PbI_2_ and SnI_2_. The calculations revealed that the binding energy significantly increases to around −2.30 eV after the loss of a proton.

### HTM Influence on perovskite Film

**Figure 3:**
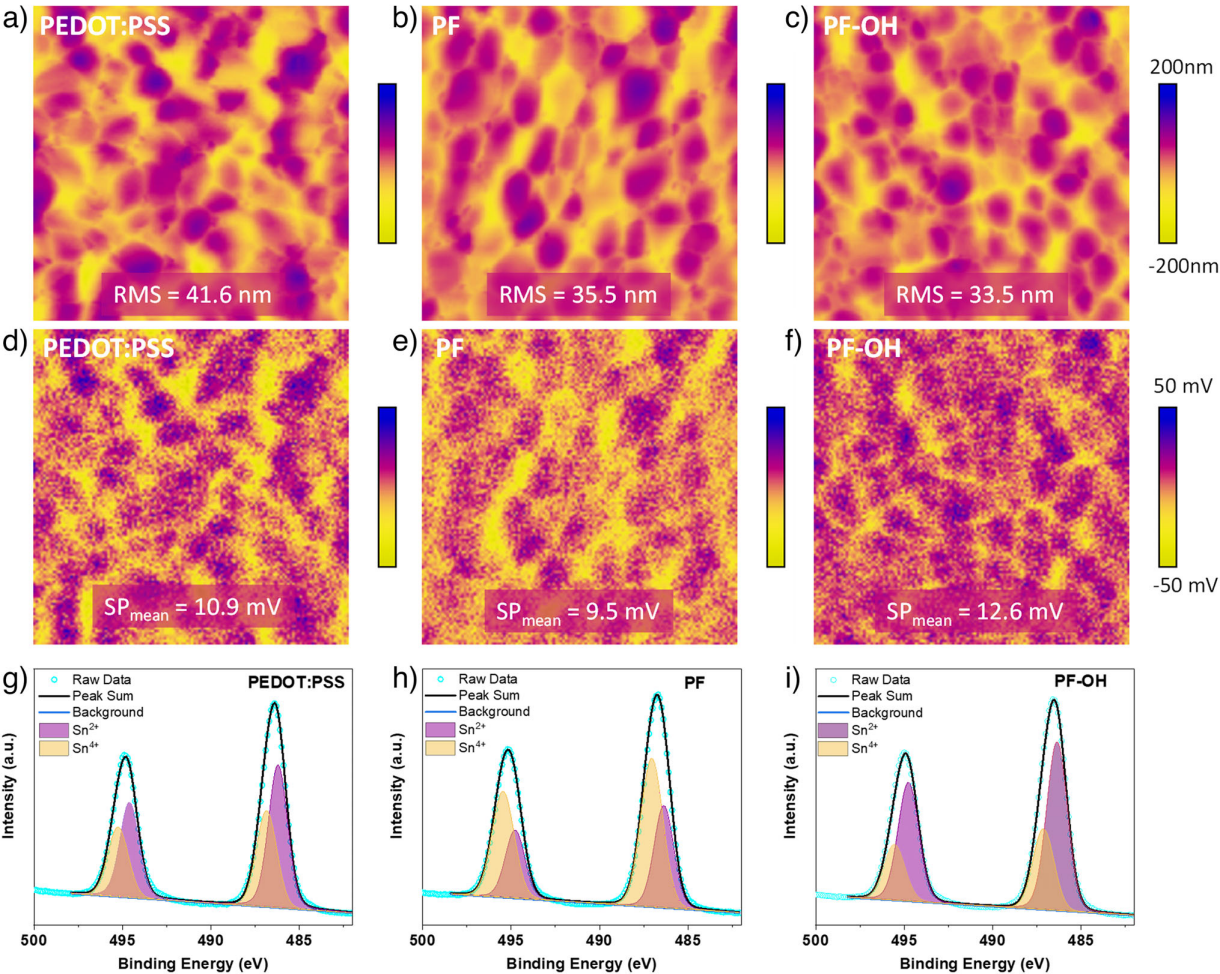
Surface morphology and Sn^4+^ distribution. AFM images of perovskite films grown on PEDOT:PSS a) PF, b) and PF─OH. c) Root mean square (RMS) and color bar indicate the roughness of perovskite films. The scanning area is 5 × 5 µm^2^. d–f) KPFM images of perovskite films. SP_mean_ and color bar, surface potential of perovskite films. The scanning area is 5 × 5 µm^2^. XPS spectra of perovskite films deposited on PEDOT:PSS g), PF h), and PF─OH i). XPS characteristic peaks can be fitted with two peaks with a mixed Lorentzian/Gaussian line shape and a linear background.

To assess the impact of HTMs on the growth of perovskites, X‐ray diffraction spectroscopy (XRD) of perovskite films grown on different substrates was conducted (Figure 2c). The perovskite films with PEDOT:PSS, PF, and PF─OH exhibit a pure perovskite phase, while perovskite film based on PF─OH shows higher crystallinity and crystallographic orientation. The enhanced film quality is further confirmed by stronger photoluminescence (PL) intensity and longer decay time observed in steady‐state and time‐resolved photoluminescence (TRPL) measurements (Figure S13). Interestingly, scanning electron microscopy (SEM, Figure 2d) and atomic force microscopy ([AFM], Figure 3a–c) revealed similar morphology and crystal size distributions. We hypothesize that the observed improvements in film quality, as indicated by the PL and TRPL data, may originate partly from an improved perovskite quality, specifically at the HTM/perovskite buried interface. To more directly observe the differences between the top and buried surfaces, we tore off the perovskite film from the substrate and measured the buried surface morphology using SEM (Figure 2e). As expected, significant differences were observed at the buried interfaces. Samples using PEDOT:PSS showed many pinholes at the grain boundaries, along with numerous rod‐like crystals. In contrast, while devoid of rod‐like crystals, PF samples exhibit numerous pinholes both on the crystals and at the grain boundaries. The PF─OH samples stand out distinctly, featuring larger crystal grains and pinhole‐free buried interfaces. This explains the discrepancy between the PL data and the SEM results, indicating that the improvement in the film quality primarily originates from the improved buried interface.

To further characterize the impact of the different HTMs on Sn─Pb perovskite films and the influence of Sn^2+^ within these films, Kelvin probe force microscopy ([KPFM], Figure 3d–f) was employed to assess the differences in surface potential, which correlates with the work function relative to the vacuum energy level. The KPFM image of the PEDOT:PSS‐based perovskite film shows notable dark areas within individual grains, indicating a heterogeneous distribution of the surface potential, whereas the perovskite film on PF─OH exhibited a more uniform surface potential distribution. The higher surface potential of the PF─OH/perovskite layer suggests a lower work function than that of PEDOT:PSS and PF‐based films, indicating a decreased self‐*p*‐doping due to the suppression of Sn^2+^ oxidation to Sn^4+^. X‐ray photoelectron spectroscopy (XPS) measurements further corroborated the decreased Sn^4+^ content in the PF─OH/perovskite film (Figure 3g–i), highlighting the role of PF─OH in effectively inhibiting the oxidation of Sn^2^⁺. This is primarily attributed to the retardation of the oxidation process through strong interactions between ─OH groups and Sn^2+^, leading to a reduced Sn vacancy density and lower hole density in Sn─Pb perovskite films.

Based on the analysis above, we propose the following interaction mechanism between PF─OH and Sn─Pb perovskite: most PF─OH molecules remain on the FTO surface during the spin coating of the perovskite precursor. The partially exposed phenol group interacts with the perovskite, passivating the undercoordinated Pb^2+^ and Sn^2+^ in the Sn─Pb perovskite through chelation. Due to its stronger interaction with perovskites, PF─OH replaces DMSO in forming complexes with PbI_2_ and SnI_2_, thereby effectively reducing buried interfacial voids. Additionally, a small amount of PF─OH dissolved in the perovskite precursor suppresses the oxidation of Sn^2+^ both in bulk and on the surface through chelation with Sn^2+^ and its intrinsic reductive properties.

### Performance and Stability of PSCs

**Figure 4:**
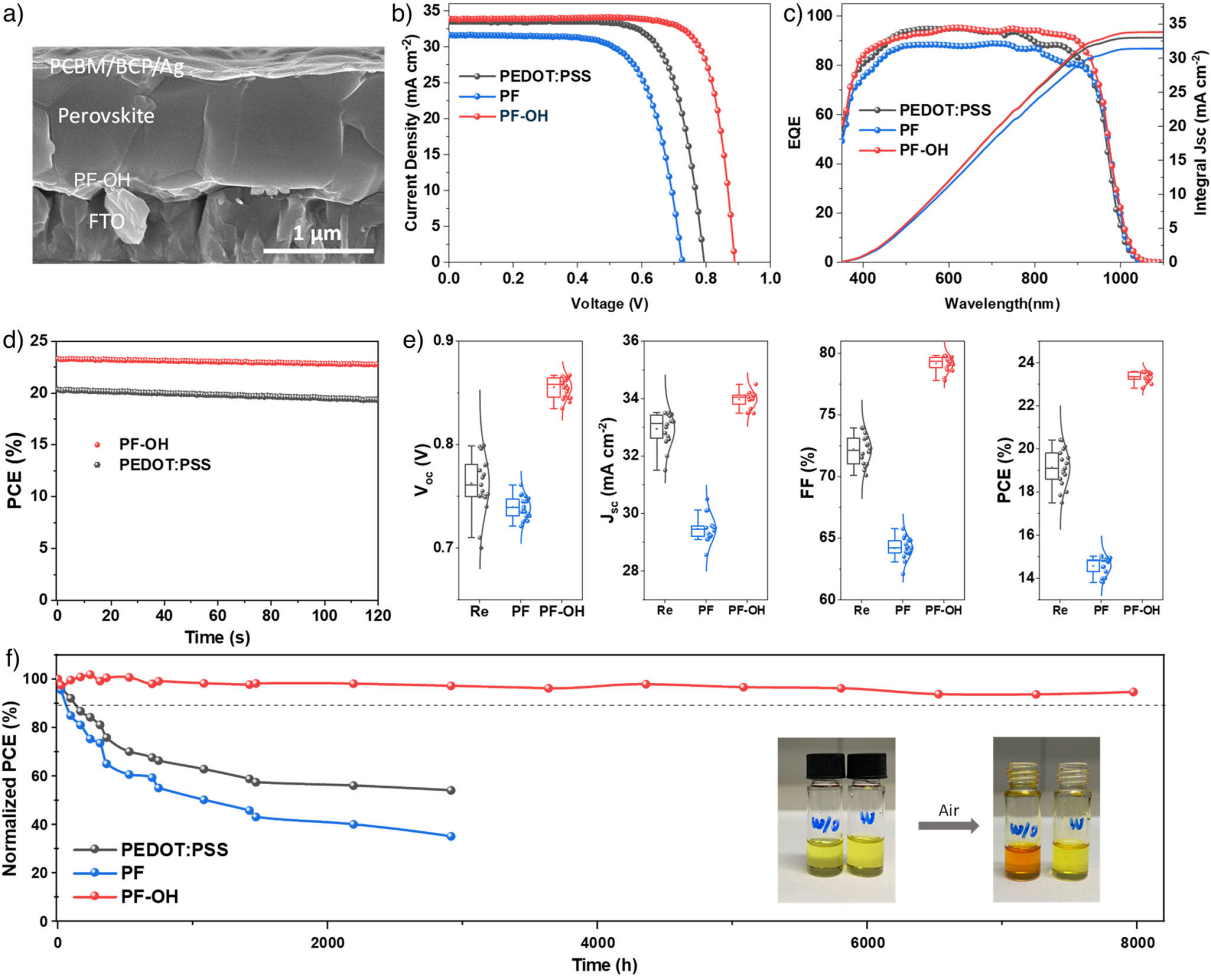
Photovoltaic performances of Sn─Pb PSCs. a) Cross‐section SEM image of PF─OH device. *J–V* curves b) and EQE c) of champion PSCs based on PEDOT:PSS, PF, and PF─OH. d) PCE of devices measured at MPP for 120 s. e) Statistical diagram of the *V*_oc_, *J*_sc_, FF, and PCE of 20 individual devices. Box plots show the 25th and 75th percentiles (box), median (line inside the box), and whiskers extending to 1.5 times the interquartile range (IQR). Outliers beyond this range are shown as individual points. f) The long‐term stability of unencapsulated cells stored in an N_2_‐filled glovebox. The inserted photographs show the ease of oxidation of Sn^2+^ to Sn^4+^ in ambient air and the inhibition of this process by PF─OH.

Having understood the antioxidative and protective properties of PF─OH, we next fabricated Sn─Pb PSCs with a structure: glass/FTO/PEDOT:PSS, PF, or PF─OH/Sn─Pb perovskite/PCBM/BCP/Ag. The cross‐sectional image of the device shows that the thickness of the Sn─Pb perovskite reaches 1.2 µm (Figure 4a), ensuring effective absorption of the incident light. From the current density–voltage (*J*–*V*) analysis in Figure 4b, the PF─OH device shows a short‐circuit current density (*J*sc) of 33.91 mA cm^−2^, an open‐circuit voltage (*V*_oc_) of 0.886, and a fill factor (FF) of 0.786, yielding a champion PCE of 23.61%. The integrated photocurrent density (33.81 mA cm^−2^) obtained from the external quantum efficiency (EQE) spectra (Figure 4c) agrees closely with the *J*_sc_ in the *J–V* curves. As a contrast, due to voids at the buried interface, all device parameters for PF significantly decrease, with a *J*_sc_ of 30.1 mA cm^−2^, a *V*_oc_ of 0.761, and an FF of 0.66, which results in a champion PCE of 15.03%. Compared to PF─OH, PEDOT:PSS mainly shows a decrease in *V*_oc_ to 0.798 and FF to 0.739, resulting in a suboptimal PCE of only 19.8%. Replacing PEDOT:PSS with PF─OH significantly improves all three photovoltaic parameters: *J*_sc_, FF, and *V*_oc_. The EQE of PF─OH shows an enhanced response in the 600–900 nm range, primarily attributed to the improved perovskite quality and enhanced transmittance of PF─OH in this region. The increase in FF can be directly correlated to the overall improved perovskite quality observed for PF─OH‐based films, as evidenced by the regulated crystallization process in the perovskite bulk, more effective defect passivation at the buried surface, and effective hole extraction at the HTM/perovskite interface. The significant improvement in *V*_oc_ with PF─OH is mainly attributed to a deeper HOMO and a better band alignment for the more intrinsic Sn─Pb perovskite.

The degradation of perovskite devices, particularly Pb‐based perovskite, is affected by multiple factors, including defects, ion migration, and interfacial contact, among others.^[^ ^39^, ^40^, ^41^ ^]^ In Sn─Pb devices, the oxidation of Sn^2^⁺ is the primary determinant of their shelf life. Conventional Sn─Pb devices typically degrade to 85% of their initial performance after just 200 h of air exposure (Figure 4f). However, the shelf stability of the PF─OH‐based devices is especially impressive, maintaining 94% of their performance even after 8000 h—around 100 times longer than that of PEDOT:PSS‐based devices, as shown in Figure 4f. This is primarily due to the improved perovskite film crystal quality and defect passivation provided by PF─OH, as well as its reductive properties. We observed that the as‐prepared yellow SnI_2_ precursor solution in mixed DMF‐DMSO solvent rapidly turned orange‐red upon exposure to air, indicating Sn^2+^ oxidation to Sn^4+^ in the precursor solution. However, the mixture containing PF─OH solution maintained the original yellow color of SnI_2_ for a long time, suggesting that PF─OH effectively suppresses the oxidation of Sn^2+^, thereby enhancing stability.

To further investigate the universality of this molecular strategy, we performed phenolic hydroxylation on the small molecule Spiro‐OMeTAD and the polymer P1,^[^ ^29^ ^]^ obtaining molecules Spiro‐OH^[^ ^34^ ^]^ and P1‐OH, respectively (Figures S19–S24). Similar to PF─OH, Spiro‐OH, and P1‐OH also demonstrated varying degrees of inhibition of Sn^2+^ oxidation when mixed into a SnI_2_ solution. Correspondingly, improvements in device stability were observed, confirming the universal applicability of phenol hydroxylation in HTMs for stabilizing Sn‐based perovskite devices.

Subsequently, the steady‐state output performance of the device (Figure 4d) was tested under AM 1.5 G at a fixed bias of the maximum power point (MPP). Compared to the device with PEDOT:PSS, the PF─OH‐based device exhibited a more stable photocurrent output and a PCE of 23.30% within 120 s. This improvement is attributed to PF─OH's antioxidation properties, reduced interfacial non‐radiative recombination loss, and improved perovskite quality. Furthermore, devices using PF─OH showed a more concentrated distribution of performance parameters (Figure 4e) due to the more uniform film formation achieved through enhanced wettability.

We further performed MPP tracking of encapsulated devices in an inert gas atmosphere. The PF─OH cell maintained 88% of its initial efficiency after 120 h of continuous operation under 1 Sun illumination at the MPP, while the PEDOT:PSS cell had a much shorter lifetime of 25 h (Figure S18). This demonstrates that PF─OH enhances the device's operational stability, likely due to suppressed non‐radiative interfacial losses in the Sn─Pb perovskite film quality and effective defect passivation.

## Conclusion

In this work, we demonstrate a multi‐functional design of a novel hole transport material that is designed around the anti‐oxidative properties of phenol substitution. Specifically, we demonstrate that phenol groups can significantly suppress the oxidation of Sn‐based perovskites. PF─OH is developed by incorporating several key principles, including integrating a multifunctional phenol group and fluorine atoms. These features endow the hole‐transporting layer with multiple functionalities: preventing Sn^2+^ oxidation, tuning interfacial energy levels, passivating surface defects, and regulating perovskite growth. As a result, the shelf stability of the devices increased by around 100 times, and the device performance also significantly improved, achieving a champion PCE of 23.61%. This work offers a general and effective molecular design strategy for developing HTMs that target highly stable and high‐performance Sn‐based perovskite cells.

## Author Contributions

J.W., J.H., S.I.S., and C.J.B. conceived the idea. S.I.S., J.H., and C.J.B. supervised the project. J.W. and M.H. wrote the manuscript. J.W. and E.A.A. performed the organic synthesis. M.H. fabricated the perovskite devices and characterized the devices. A.B. measured the SEM. J.Y., C.L., and G.F.H. analyzed the data. Q.D., T.W., and Q.J. performed DFT calculations. All authors reviewed and edited the manuscript.

## Conflict of Interests

The authors declare no conflict of interest.

## Supporting information



Supporting Information

## Data Availability

The data that support the findings of this study are available in the Supporting Information of this article.
