# Supplementary material for: Highly Stable Sn─Pb Perovskite Solar Cells Enabled by Phenol‐Functionalized Hole Transporting Material
Source: Angew Chem Int Ed Engl. 2025 Apr 2;64(22):e202424515. doi: 10.1002/anie.202424515 (PMC12105703; doi:10.1002/anie.202424515)
Supplement: Supplementary file 1 — Supporting Information [file ANIE-64-e202424515-s001.docx]

Supporting Information

Highly Stable Sn-Pb Perovskite Solar Cells Enabled by Phenol-Functionalized Hole Transporting Material
Jianchang Wu et al.

**The PDF file includes:**Materials and Methods
Figure. S1 to S24

**Materials and Methods
Materials**

Reagents and solvents for organic synthesis were purchased from commercial suppliers (Fluorochem, Sigma-Aldrich, BLD pharm, TCI Europe) and used with no further purification unless otherwise noted. Thin layer chromatography (TLC) plates were purchased from Sigma-Aldrich.

Chemicals for perovskite solar cells: Formamidinium iodide (FAI), Methylammonium iodide (MAI) were purchased from Greatcell Solar Materials. lead iodide (PbI_2_), and were purchased from TCI chemicals. Cesium iodide (CsI), *N,N*-Dimethylformamide (DMF), isopropyl alcohol, dimethyl sulfoxide (DMSO), chlorobenzene (CB), toluene (TL), isopropanol (IPA, 99.5%), tin(II) iodide (SnI_2_, 99.999%), tin(II) fluorine (SnF_2_, 99%), [6,6]-Phenyl C_61_ butyric acid methyl ester (PCBM), and bathocuproine (BCP) were purchased from Sigma-Aldrich. PEDOT:PSS (CLEVIOS PVP AI 4083) was obtained from Heraeus. Ethylenediammonium diiodide (EDAI_2_, 98%) were purchased from Xi’an Polymer Light Technology.

**Synthesis of 4,4'-(9-(2,3,5,6-tetrafluoro-4-vinylphenyl)-9H-carbazole-3,6-diyl)bis(N,N-bis(4-methoxyphenyl)aniline)**

To a solution of 6 (1.0 g, 1.29 mmol) in dry DMF (10 mL) was added 60% NaH oil (77 mg, 1.94 mmol). The mixture was stirred at room temperature for 1 h. Pentafluorostyrene (0.30 g, 1.55 mmol) was added to the above solution by syringe. The mixture was heated at 60 °C for 24 h. Water was added to quench the reaction. The organic solvent was extracted with CH_2_Cl_2_ and water to remove DMF. After the removal of CH_2_Cl_2_, the residue was directly purified by column chromatography on silica gel (petroleum ether/CH_2_Cl_2_ = 1:1) to give a white solid (1.15 g, with a yield of 94%). ^1^H NMR (400 MHz, DMSO-d6) δ 8.60 (s, 2H), 7.72 (d, J = 8.6 Hz, 2H), 7.64 (d, J = 8.6 Hz, 4H), 7.43 (d, J = 8.4 Hz, 2H), 7.06 (d, J = 8.9 Hz, 8H), 6.96 – 6.88 (m, 13H), 6.22 (d, J = 18.0 Hz, 1H), 5.97 (d, J = 11.8 Hz, 1H), 3.75 (s, 12H). ^13^C NMR (100 MHz, DMSO) δ 156.1, 147.9, 146.5, 145.2, 144.1, 142.8, 140.7, 139.4, 134.1, 132.9, 127.9, 126.9, 125.7, 124.6, 122.4, 120.6, 118.7, 116.9, 115.4, 111.1, 55.7. ^19^F NMR (376 MHz, DMSO-*d_6_*) δ -142.97 , -146.02 . HRMS (ESI): m/z [M]+calcd for C_60_H_46_F_4_N_3_O_4_, 948.0306; found, 948.3412.

**Synthesis of PF**

The monomer 2 (0.53 g, 0.56 mmol) was polymerized in THF (1.5 mL) with 1 wt % AIBN (5.3 mg) as the initiator under nitrogen at 85 °C for 2 days. The polymerization was stopped by pouring the reaction mixture into methanol. The obtained yellow polymer was purified by repeated reprecipitation from methanol followed by drying under vacuum (0.45 g, with a yield of 85%). Mn = 2.06 × 10^4^; Mw/Mn = 1.61.

**Synthesis of PF-OH**

A solution of PF (1 eq.) in dry CH_2_Cl_2_ (20 ml) was cooled to -70 °C by liquid N2 and ethanol, and then BBr_3_ (10 eq.) was added dropwise under N2. After 30 min, the cold bath was removed and the mixture war stirred at room temperature for 1 day. Methanol (15 mL) was slowly added over 15 min and then the solvent was evaporated. The mixtures were purified by toluene washing. Mn = 1.83 × 10^4^; Mw/Mn = 1.57. P1-OH and Spiro-OH were synthesized using the same way.

**Perovskite precursor preparation**

1.8M FA_0.7_MA_0.3_Pb_0.5_Sn_0.5_I_3_ precursor was prepared by dissolving 1.26 mmol FAI, 0.54 mmol MAI, 0.90 mmol PbI_2_, 0.90 mmol SnI_2_, 0.09 mmol SnF_2_ and 0.1 mm% TACl in 1 ml DMF:DMSO (3:1, v/v) and stirred overnight. Tin powders (5 mg/ml) were added in the precursor and stirred at room temperature for 20 min before use. The precursor solution with the remaining tin powders was filtered through 0.20-μm PTFE membrane before the perovskite films were made.

**Pb–Sn narrow-bandgap PSC fabrication**

The pre-patterned FTO glass substrates were sequentially cleaned using acetone and isopropanol. For reference, PEDOT:PSS (Al 4083) was diluted 5 times with methanol, then spin-coated on FTO substrates at 4,000 r.p.m. for 30 s and annealed on a hotplate at 150 °C for 10 min in ambient air. After cooling, the substrates were transfer immediately to a nitrogen-filled glovebox for the deposition of perovskite films. For PF and PF-OH, they are dissolved in chlorobenzene and isopropyl alcohol (IPA) with 0.5-5 mg/ml, respectively . Then spin-coated on FTO substrates at 5,000 r.p.m. for 30 s and annealed on a hotplate at 100 °C for 10 min in glovebox. The perovskite films were deposited with two-step spin-coating procedures: (1) 1,000 r.p.m. for 10 s with an acceleration of 200 r.p.m. s^–1^ and (2) 4,000 r.p.m. for 40 s with a ramp-up of 1,000 r.p.m. s^–1^. Chlorobenzene (500µl) was dropped on the spinning substrate during the second spin-coating step at 20 s before the end of the procedure. The substrates were then transferred to a hotplate and heated at 100 °C for 10 min. PCBM solution (20 mg/ml in chlorobenzene) is spin-coated on top of perovskite at 1000rpm for 30s, then annealed at 80°C for 10 min. BCP solution (0.5 mg/ml in isopropanol) is spin-coated on top of PCBM at 5000rpm for 30s, annealed 80°C for 5 min. After cooling down to room temperature, the substrates were transferred to the evaporation system. 100 nm thick Ag layer was thermally evaporated under a vacuum of 8×10^−6^ mbar at a rate of ∼0.1 nm/s to finish the device fabrication.

**Characterization**

The *J-V* curves were measured using a Keithley 2400 source meter and a solar simulator (Newport, Oriel Class A, 91195A) producing 100 mW·cm^−2^ illumination (AM 1.5 G) under air atmosphere. The unencapsulated devices were measured both in reverse [forward bias (1.5 V) → short circuit (0 V)] or forward [short circuit (0 V) → forward bias (1.5 V)] scan modes. The step voltage and delay time were fixed at 0.05 V and 100 ms, respectively. The device area was 0.18 cm^2^ and marked by aperture shade mask to define active area of 0.096 cm^2^. External quantum efficiency (EQE) was measured using an internal quantum efficiency system (Oriel, IQE 200B) under irradiation by a 100 W Xenon lamp. During the storage stability test, the devices were stored in a nitrogen-filled glovebox. For each measurement, they were taken out and tested in ambient air. In the first two weeks, measurements were conducted daily, and thereafter, every two days. During each testing session, the devices were exposed to air for approximately 30 min. The x-ray photoelectron spectroscopy (XPS) analyses were carried out using Thermo Fisher Scientific (ESCALAB 250XI). Film morphologies were measured using a scanning electron microscope (Cold FE-SEM SU-8220). The optical properties of the films were measured using UV-Vis spectroscopy (Shimadzu UV-2600). Time-resolved photoluminescence (TRPL) spectra were recorded using a commercial time-correlated single photon counting (TCSPC) setup (FluoTime 300, PicoQuant GmbH) equipped with a PMA-C-192-M detector and high-resolution excitation monochromators. Differential scanning calorimetry (DSC) measurements were performed using TA (Q200). The hydrophobicity of each HTM was measured using contact angle equipment (Phoenix 300, SEO). NMR spectra were recorded on a Brucker Avance III 400 MHz (and 100 MHz for 1H and 13C NMR, respectively). Chemical shifts were reported as δ values (ppm) with tetramethylsilane (TMS) as the internal standard. The splitting patterns are designated as follows: singlet (s), doublet (d), triplet (t), and multiplet (m).

**Computational method**

**Evaluation of electrostatic surface potential and dipole moment**

The repeat unit of PF or PF-OH was first optimized by density functional theory (DFT) calculations at the ωB97XD/6-31G(d,p) level of theory. Then, the corresponding electrostatic surface potential and dipole moment were evaluated at the gas-tuned-ωB97XD/6-31G(d,p) level of theory. Herein, all the DFT calculations were carried out within the Gaussian 16 package^1^.

**Evaluation of binding energies of metal iodides** **(MI_2_)-PF-O^−^, MI_2_-DMSO, and MI_2_-FAI**

The spin-unrestricted DFT calculations (towards the binding energies of MI_2_-PF-O^−^, MI_2_-DMSO, and MI_2_-FAI) were performed in the DMol3 code^2,3^. The generalized gradient approximation (GGA) with the Perdew−Burke−Ernzerhof (PBE) functional was employed to describe the exchange and correlation effects^4^. The DFT Semi-core Pseudopots (DSPP) was used for core treatment, where the core electrons are replaced by a single effective potential and some degree of relativistic correction is introduced into the core^5^. A smearing of 0.005 Ha (1 Ha = 27.21 eV) to the orbital occupation was applied to achieve accurate electronic convergence. The TS method was used for the DFT-D correction^6^. For geometry optimization, the convergence tolerances of energy, maximum force, and displacement were set as 1.0×10^-5^ Ha, 0.002 Ha/Å, and 0.005 Å, respectively. The binding energies, *E*_bi_, of PF-O^−^, DMSO, and FAI with MI_2_ were calculated by the following equation: *E*_bi_ = *E*_bisys_ – *E*_mol_ – *E*_MI2_, where *E*_bisys_, *E*_mol_, and *E*_MI2_ are the energies of the binding system, the PF-O^−^, DMSO, or FAI, and the PbI_2_ or SnI_2_, respectively. The effect of DMSO on the binding energies of MI_2_-PF-O^−^ and MI_2_-FAI was considered using the solvation model (*i.e.*, conductor-like screening model, COSMO) with a dielectric constant of DMSO (46.7).

**Evaluation of adsorption of** **O_2_ on SnI_2_ with and without** **phenol**

The adsorption configurations of O_2_ on SnI_2_ or SnI_2_-phenol were optimized by spin-unrestricted DFT-D calculations at the GGA PBE level of theory, where the convergence tolerances of energy, maximum force, and displacement were set as 2.0×10^-5^ Ha, 0.004 Ha/Å, and 0.005 Å, respectively. The adsorption energies, *E*_ad_, of O_2_ on SnI_2_ or SnI_2_-phenol were calculated by: *E*_ad_ = *E*_adsys_ – *E*_O2_ – *E*_SnI2_, where *E*_adsys_, *E*_O2_, and *E*_SnI2_ are the energies of the adsorption system, the O_2_, and the SnI_2_ or SnI_2_-phenol, respectively.

**Evaluation of** **SnI_2_ oxidation process and reaction energy barrier**

The search of transition states (TS) for SnI_2_ oxidation process was carried out by spin-unrestricted DFT-D calculations at the GGA PBE level, before which the reactants and products were optimized. The complete LST/QST was adopted as the search protocol, where the LST (linear synchronous transit) was first performed and then followed by the repeated conjugate gradient minimizations and QST (quadratic synchronous transit) maximizations until a TS was located.

**Evaluation of binding energies between PF, PF-OH, or PF-O****^−^ and PbI_2_ or SnI_2_**

The corresponding DFT-D calculations followed the procedure given in the evaluation of binding energies of MI_2_-PF-O^−^, MI_2_-DMSO, and MI_2_-FAI, where no DMSO effect was considered.

**Evaluation of adsorption energies of PF or PF-OH on** **FA_0.7_MA_0.3_Pb_0.5_Sn_0.5_I_3_ surface**

Based on the FAPbI_3_ 2×4×4 supercell, the bulk FA_0.7_MA_0.3_Pb_0.5_Sn_0.5_I_3_ was first modeled approximately by replacing half of the Pb atoms with Sn atoms and 5/16 FAs with MAs. Then, the bulk FA_0.7_MA_0.3_Pb_0.5_Sn_0.5_I_3_ was optimized by spin-unrestricted DFT-D calculations at the GGA PBE level. Following this, the FA_0.7_MA_0.3_Pb_0.5_Sn_0.5_I_3_ (100) surface was built adding a 50 Å vacuum along the surface normal direction. Finally, the PF or PF was placed on the FA_0.7_MA_0.3_Pb_0.5_Sn_0.5_I_3_ (100) surface with the F atom close to the Sn and Pb atoms, respectively. For geometry optimization, the convergence tolerances of energy, maximum force, and displacement were set as 2.0×10^-5^ Ha, 0.004 Ha/Å, and 0.005 Å, respectively. The Brillouin zone sampling was performed with 1×1×1 k-points meshes. The adsorption energy, *E*_ads_, of the relevant adsorbate on the FA_0.7_MA_0.3_Pb_0.5_Sn_0.5_I_3_ (100) surface was calculated by the following equation: *E*_ad_ = *E*_adsys_ – *E*_surface_ – *E*_adsorbate_, where *E*_adsys_, *E*_surface_, and *E*_adsorbate_ are the total energies of the adsorption system, the modeled FA_0.7_MA_0.3_Pb_0.5_Sn_0.5_I_3_ (100) surface, and the relevant adsorbate of PF or PF-OH, respectively. Herein, all the DFT calculations were performed in the DMol^3^ code^2,3^.

**Figure S1.** O_2_ adsorption and O_2_-phenol co-adsorption on SnI_2_.

**Figure S2.** SnI_2_ oxidation process and reaction energy barrier.

**Figure S3.** SnI_2_ oxidation process and reaction energy barrier in the presence of phenol.

**Figure S4.** Electrostatic surface potential and dipole moment of PF, PF-OH, and PF-O-. PF-O- is the product formed after proton transfer in PF-OH or its reaction with a basic reagent, like FAI.

**Figure S5. a**. UV-vis absorption of PF in THF and PF-OH in IPA. **b**. J^1/2^–V characteristics based on hole only devices: ITO/PEDOT:PSS/PF or PF-OH/MoO_3_/Ag. **c**. cyclic voltammograms.


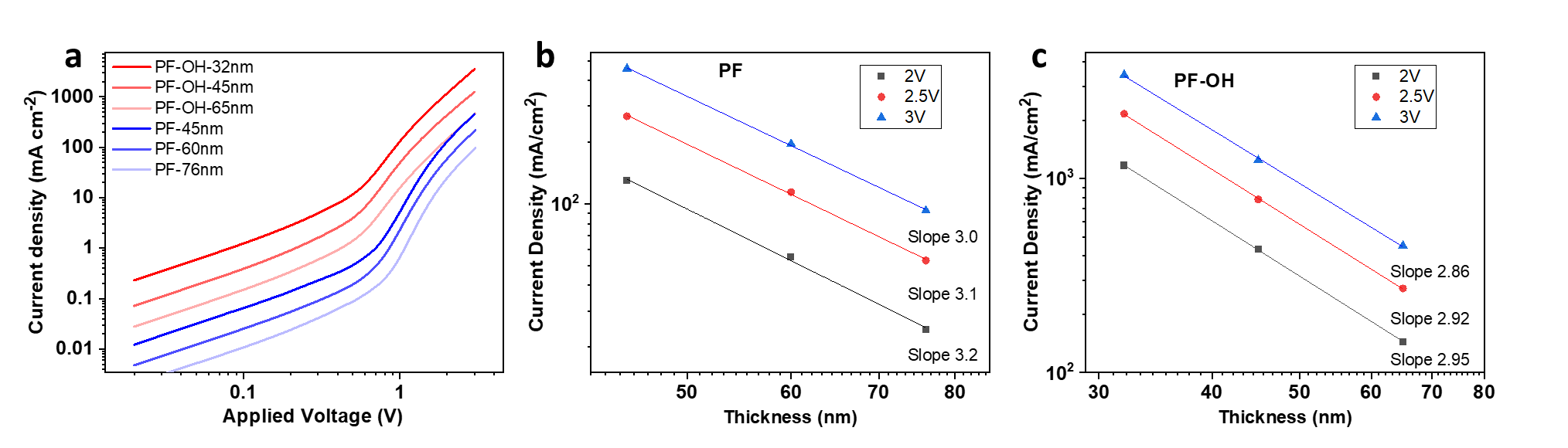


**Figure S6. a.** Thickness-dependent SCLC of PF and PF-OH based hole-only device. **b-c.** Linear correlations of lg(J) and lg(L) with a slope close to 3 confirming the space limited charge current when the voltage is higher than 2 V.

**Figure S7.** DSC of PF and PF-OH.


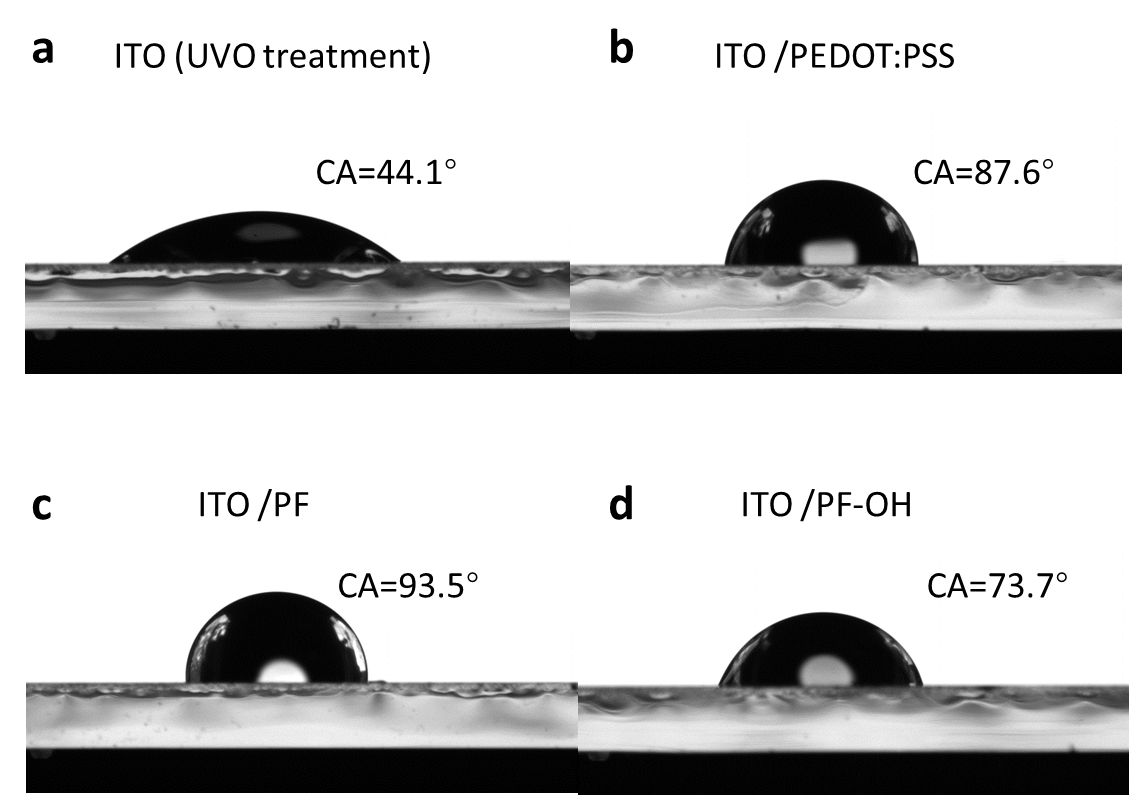


**Figure S8.** Contact angle of (**a**) ITO, (**b**) ITO/PEDOT:PSS, (**c**) ITO/PF, (**d**) ITO/PF-OH.

**Figure S9.** Transmittance spectra of FTO/PEDOT:PSS, and FTO/PF-OH.

**Figure S10.** UV-Vis absorption of PF-OH film before and after washing with DMF:DMSO mixed solvent. To more clearly observe the effect of the solvent on cleaning the film, we prepared a thick film using a high-concentration solution (10mg/ml in IPA).

**Figure S11.** ^1^H NMR of MAI, FAI and mixture of PF-OH/perovskite powder scraped from FTO glass in DMSO-*d6*.

**Figure S12.** Binding energies of PF, PF-OH with SnI_2_ and PbI_2_, respectively.

**Figure S13.** PL (**a**), and TRPL decays (**b**) of perovskite films grow on different HTMs.

**Figure S14.** Models of the interactions between PF, PF-OH and PF-O^-^ with PbI_2_ and SnI_2_.

**Figure S15.** Models and sites for interaction between undercoordinated Pb^2+^ and Sn^2+^ with PF and PF-OH.

**Figure S16.** *J-V* curves of device based on PF-OH with concentration from 0.5 mg/ml to 4 mg/ml in IPA.

**Figure S17.** *V_OC_* dependence on light intensity of devices based on PEDOT:PSS and PF-OH.

**Figure S18.** MPP tracking of devices based on PEDOT:PSS, PF, and PF-OH.


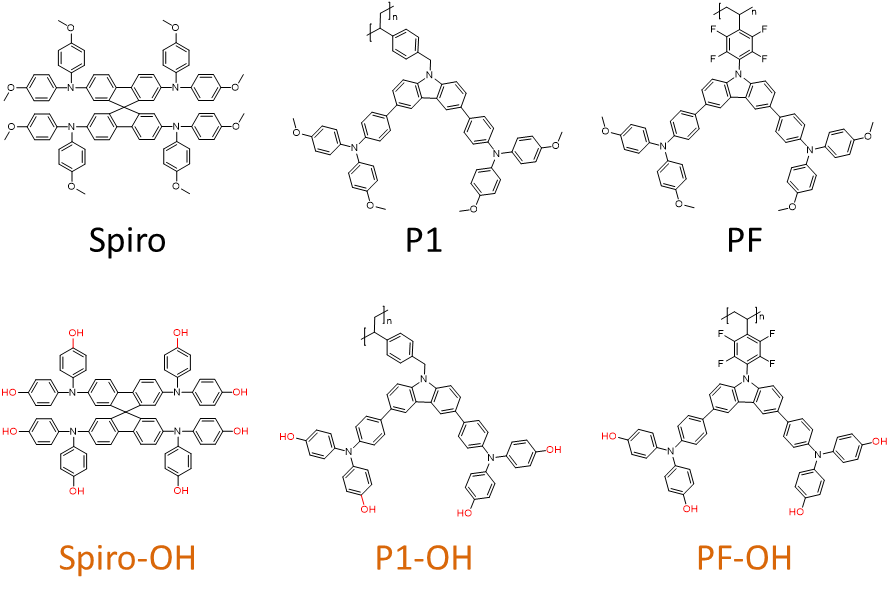


**Figure S19**. Chemical structures of HTMs for Pb-sn perovskite device.


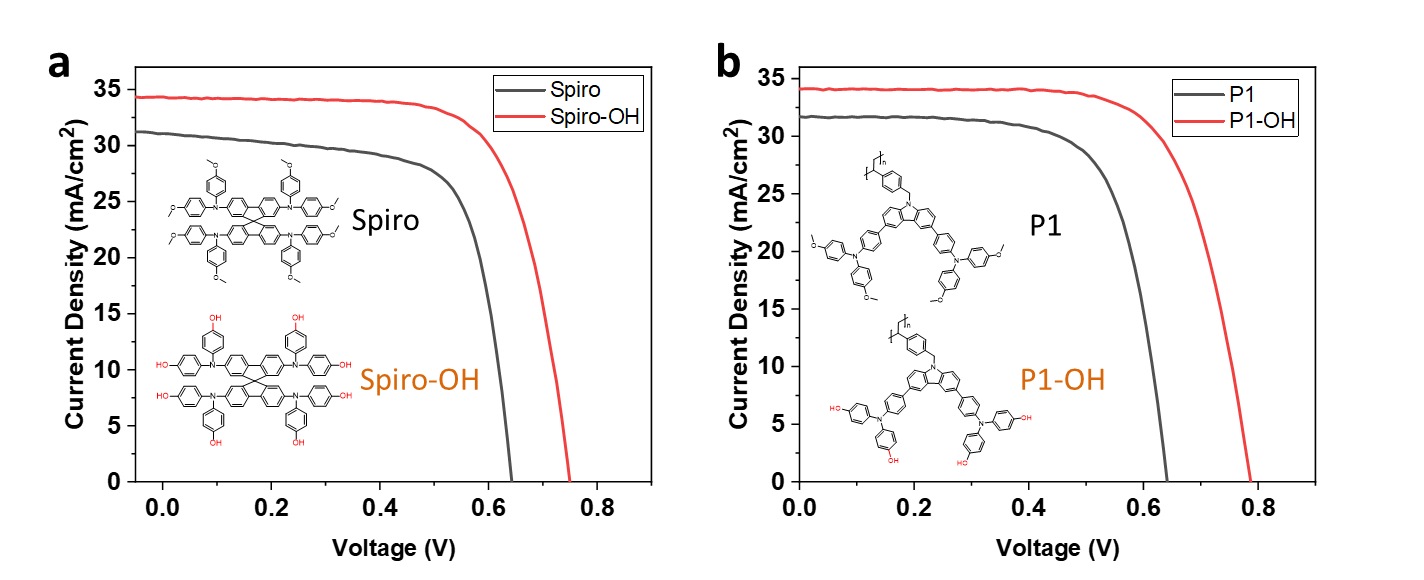


**Figure S20**. *J-V* curves of pb-sn device based on spiro, spiro-OH (**a**), P1 and P1-OH (**b**).


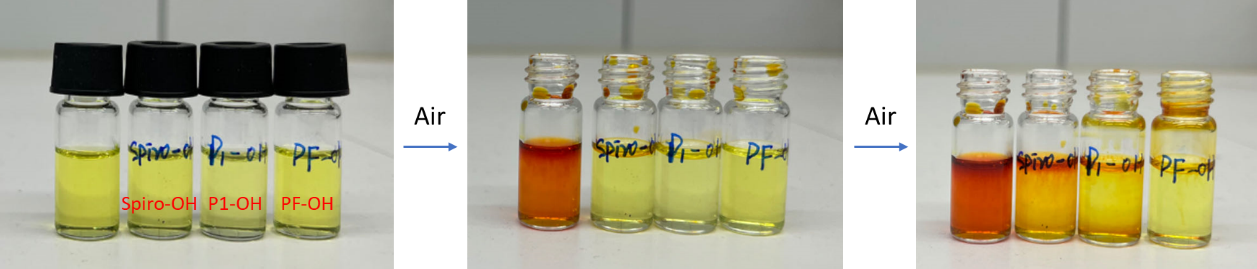


**Figure S21**. Oxidation process of SnI_2_ solution mixed with different materials.


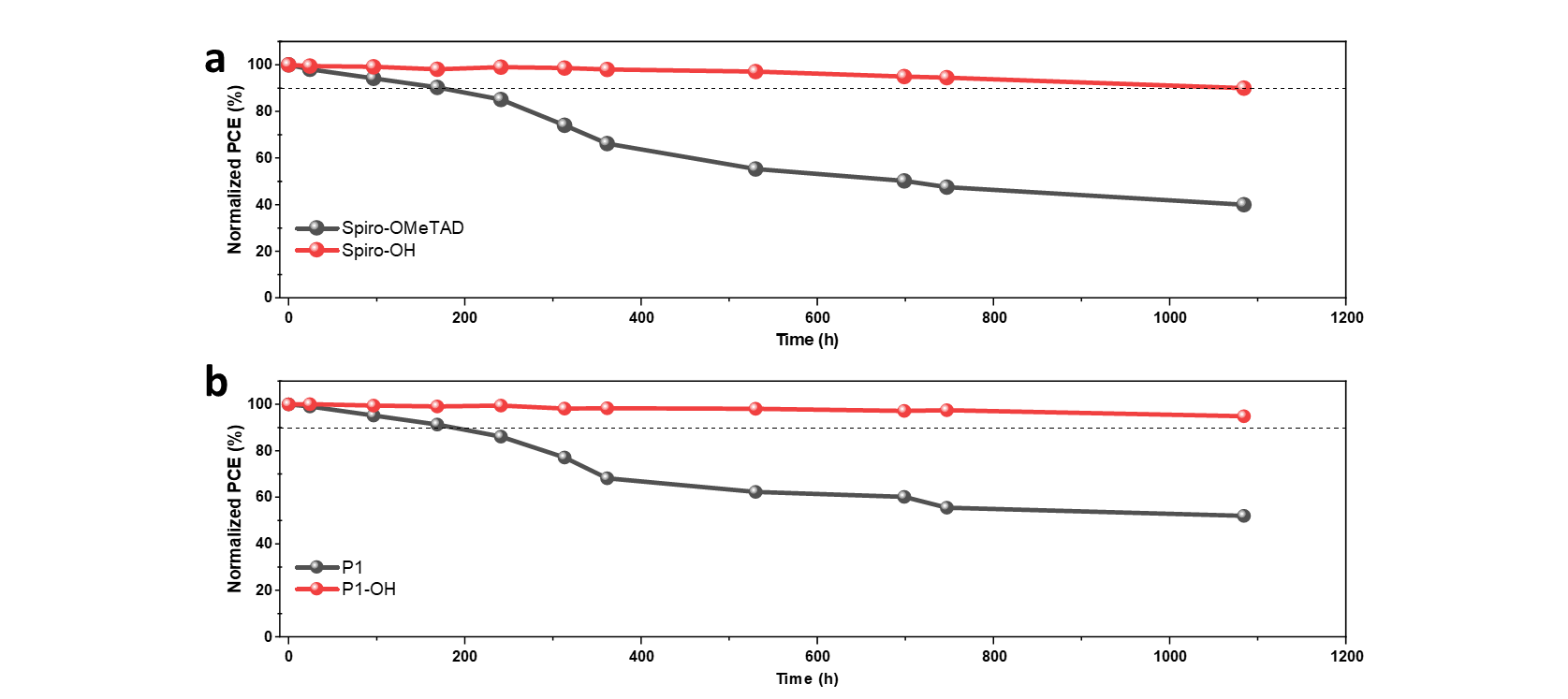


**Figure S22**. The long-term stability of unencapsulated cells stored in an N2-filled glovebox.


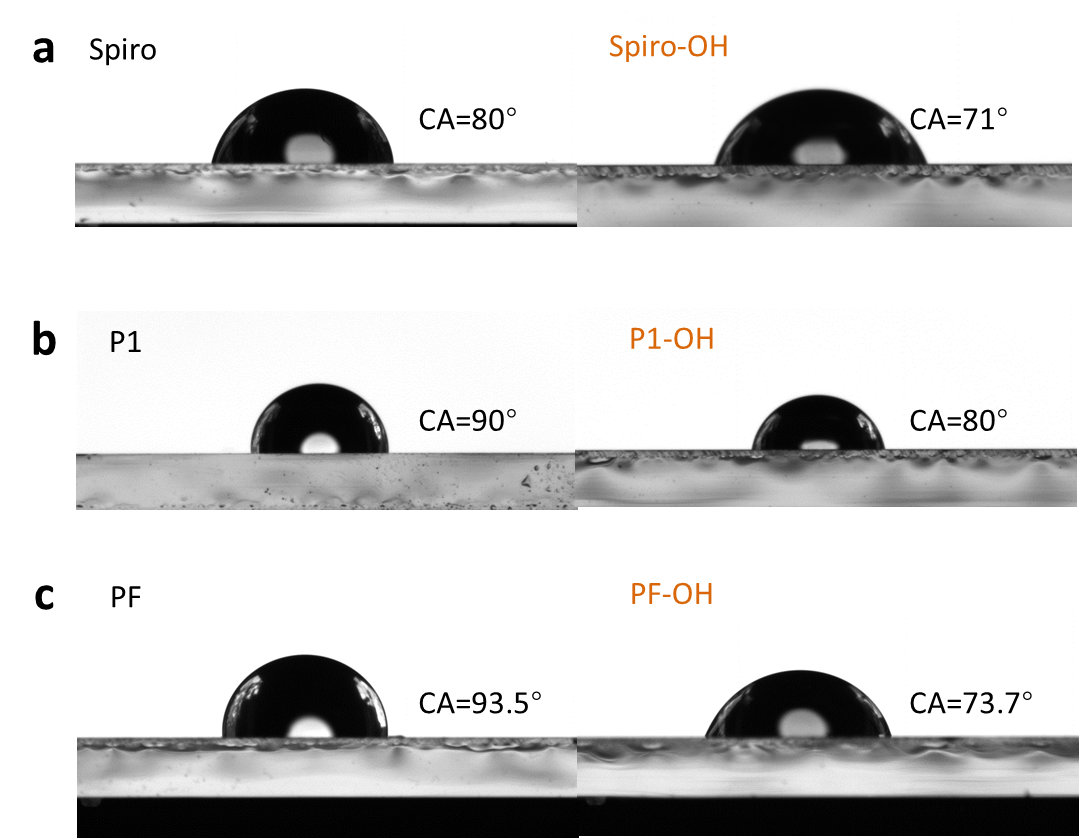


**Figure S23**. Contact angle of (**a**) Spiro-OMeTAD and Spiro-OH, (**b**) P1 and P1-OH, (**c**) PF and PF-OH.


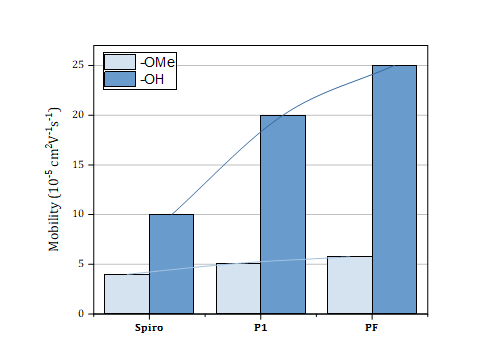


**Figure S24**. SCLC Mobilities of molecules.


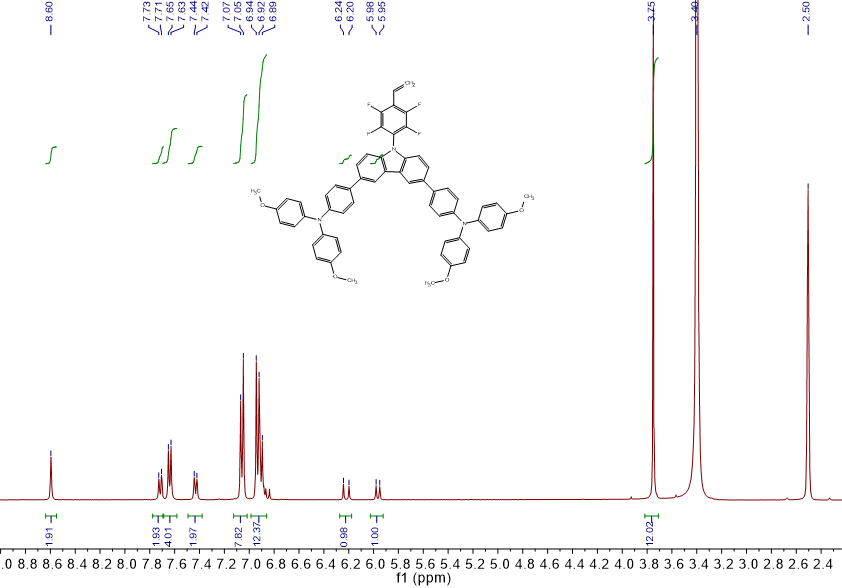


^1^H NMR of monomer 2 in DMSO-*d6*.

^13^C NMR of monomer 2 in DMSO-*d6*.

^19^F NMR of monomer 2 in DMSO-*d6*.


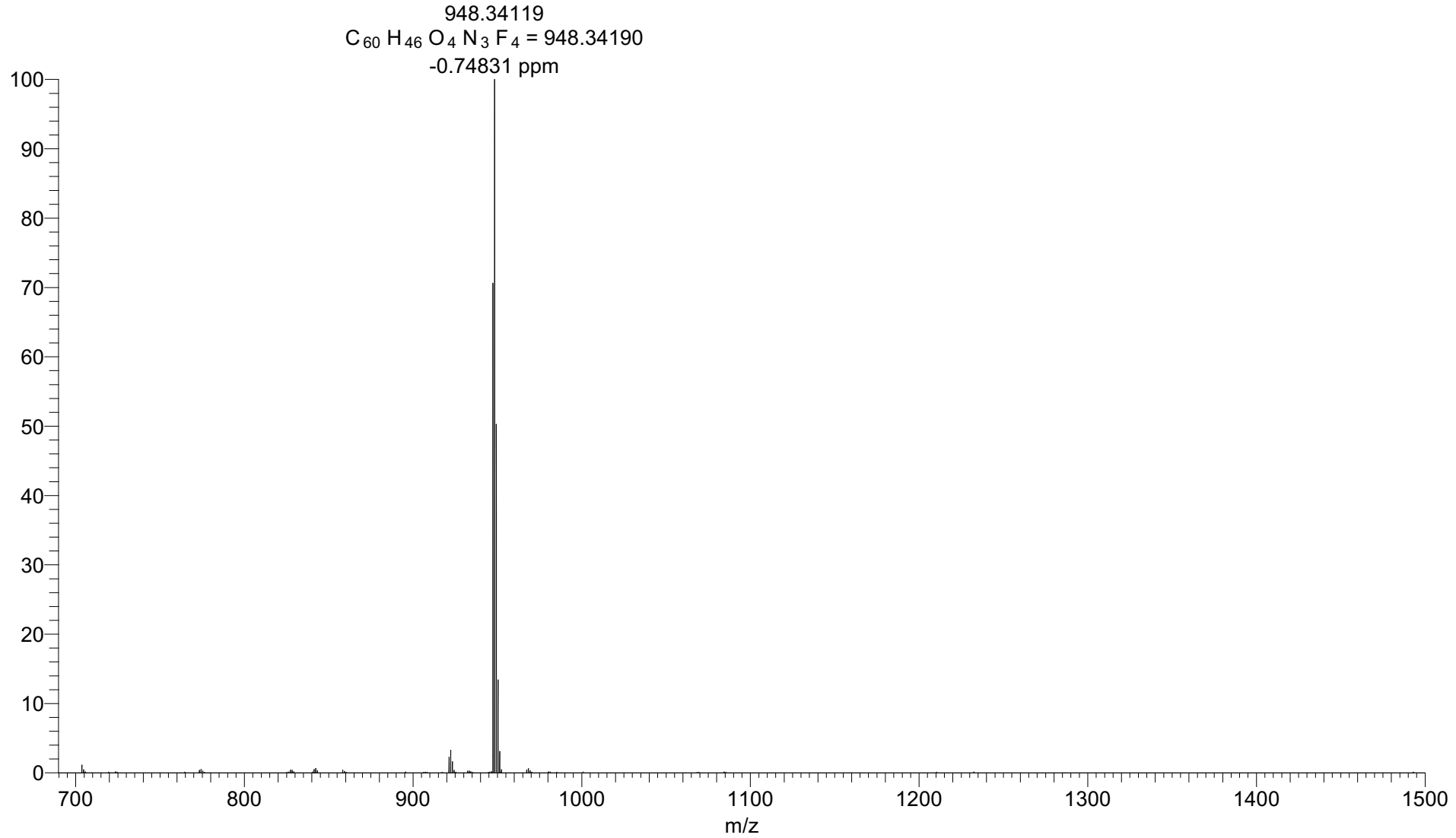


HR MS of monomer 2.

References

1 Gaussian 16 Rev. C.01 (Wallingford, CT, 2016).

2 Delley, B. *J. Chem. Phys.* **92**, 508 (1990).

3 Delley, B. *J. Chem. Phys.* **113**, 7756 (2000).

4 Perdew, J. P., Burke, K. & Ernzerhof, M. *Phys. Rev. Lett.* **77**, 3865 (1996).

5 Delley, B. *Phys. Rev. B* **66**, 155125 (2002).

6 Tkatchenko, A. & Scheffler, M. *Phys. Rev. Lett.* **102**, 073005 (2009).
